# Echoes of 1816: microbial footprints in heritage artifacts from Argentina’s museum of independence

**DOI:** 10.3389/fmicb.2025.1611832

**Published:** 2025-07-10

**Authors:** Daniel Gonzalo Alonso-Reyes, Fátima Silvina Galván, Natalia Noelia Alvarado, María Cecilia D’Arpino, Luciano José Martinez, Hernán José Esquivel, Cecilia Aymara Gallardo, María Julia Silva Manco, Virginia Helena Albarracín

**Affiliations:** ^1^Laboratorio de Microbiología Ultraestructural y Molecular, Centro Integral de Microscopía Electrónica (CIME), Universidad Nacional de Tucumán, CONICET NOA SUR, CONICET, Tucumán, Argentina; ^2^Facultad de Agronomía, Zootecnia y Veterinaria, Universidad Nacional de Tucumán, Tucumán, Argentina; ^3^Instituto de Salud y Calidad de Vida, Universidad de San Pablo, Tucumán, Argentina; ^4^Facultad de Ciencias Naturales e Instituto Miguel Lillo, Universidad Nacional de Tucumán, Tucumán, Argentina

**Keywords:** biodiversity, cultural heritage, museum, extremophiles, electron microscopy, Tucuman, microbiomes, biodeterioration

## Abstract

**Introduction:**

Historical artifacts preserved in museums are invaluable cultural treasures but are often susceptible to biodeterioration driven by microbial colonization. Despite increasing awareness of microbial impacts on heritage conservation, systematic microbiological studies of such objects remain limited, particularly in Latin America.

**Methods:**

This study presents the first comprehensive investigation of bacteria inhabiting heritage artifacts from Casa Histórica de la Independencia, Argentina’s Museum of Independence. Samples were collected from a range of materials, including wood, textiles, architectural elements, and exterior walls. Microbial colonization was assessed using scanning electron microscopy (SEM), and bacterial isolates were phenotypically and taxonomically characterized via VITEK MALDI-TOF mass spectrometry. Selected isolates were further subjected to genomic analysis.

**Results:**

SEM imaging revealed diverse and well-structured biofilms with intricate three-dimensional architectures embedded in extracellular polymeric substances. A total of 49 bacterial strains were isolated, predominantly Gram-positive genera such as *Bacillus*, *Micrococcus*, and *Kocuria*. The 19th-century albumen print photograph emerged as the most biodiverse artifact, yielding 21 distinct strains, including extremophilic genera such as *Streptomyces, Oceanobacillus*, and *Caldibacillus thermoamylovorans*. The albumen layer’s protein-rich and halophilic properties likely promoted microbial colonization. Notably, *Pseudomonas* species were found exclusively on this photographic material. Human-associated taxa, such as *Staphylococcus epidermidis* and *Staphylococcus equorum*, were primarily detected in high-contact zones, while exterior surfaces exhibited unique microbial profiles, including opportunistic pathogens.

**Discussion:**

The findings highlight a complex and substrate-specific microbial landscape across the museum’s collection. The presence of halophiles and human-associated bacteria points to both intrinsic material properties and external contamination sources. This study shows the importance of incorporating microbiological data into conservation protocols. By characterizing the artifact-associated microbiota, we contribute to the emerging field of heritage microbiology and future bio-informed preservation strategies.

## 1 Introduction

Museums serve a fundamental role in the conservation of cultural heritage (CH), safeguarding irreplaceable artifacts that embody humanity’s identity, memory, and history. Preventive conservation is vital because cultural assets are inherently vulnerable to deterioration processes that threaten their integrity (Gutarowska et al., 2014; Grabek-Lejko et al., 2017; Negi and Sarethy, 2019; Baldan et al., 2021; Liu et al., 2022). Microbial contamination is a common issue in heritage collections, as both organic and inorganic materials can be colonized by bacteria and fungi depending on their bioreceptivity and environmental exposure. These microorganisms persist in museum environments by adapting their versatile metabolisms to low-nutrient conditions and limited light, and by tolerating subtle microclimatic fluctuations that may occur despite environmental controls. Indeed, microbes may have been present in the original piece since its first production, as invisible part of the materials used to craft the artifact. Microbes settle into specific ecological niches, often remaining undetected until visible biofilm formation, discoloration, or structural weakening became apparent. Over time, their activity can cause significant damage to textiles, wood, paper, and other organic materials, manifesting as discoloration, structural weakening, odor, and cracking (Skóra et al., 2015; Grabek-Lejko et al., 2017; Negi and Sarethy, 2019). The consequences of microbial growth extend beyond artifact damage, potentially diminishing monetary value, and bringing about costly decontamination procedures (Sterflinger and Piñar, 2013). Despite these challenges, the cultural and historical value of many CH objects is immeasurable and cannot be quantified solely in monetary terms.

Biodeterioration is defined as “any undesirable change in a material brought about by the vital activities of organisms” (Perito and Cavalieri, 2018). While biodeterioration cannot be entirely halted, advances in microbial ecology and technological interventions offer strategies to mitigate its effects. A deeper understanding of microbial communities on CH objects is critical for identifying their deteriorative mechanisms and implementing effective control measures (Perito and Cavalieri, 2018; Baldan et al., 2021). These aspects have become an important research field for both, conservators and microbiologists (Adamiak et al., 2018). This issue also raises occupational health concerns, as museum staff may be exposed to harmful biological agents because of the quality of the indoor air (Baldan et al., 2021), or during the handling of contaminated materials, such as paper, wood, and textiles (Borrego et al., 2010; Gutarowska et al., 2014; Stratigaki et al., 2024). Indeed, many studies indicated that conservators, librarians, and archivists face risks related to microbial exposure (Zielińska-Jankiewicz et al., 2008; Wiszniewska et al., 2009; Karbowska-Berent et al., 2011; Baldan et al., 2021).

Traditional methods of investigating microorganisms on CH objects have primarily been culture-dependent, offering insights into their deteriorative roles. Recently, amplicon sequencing has emerged as a powerful yet resource-intensive tool for microbial identification (Kraková et al., 2012a, 2018). An alternative approach could be the combination of the automated VITEK system for rapid biochemical identification (Książczyk et al., 2016), with whole-genome sequencing (WGS) for functional profiling of key strains. Scanning electron microscopy (SEM) has also proven effective in biodeterioration studies, providing visual insights into microbial activity on CH surfaces (Díaz-Herráiz, 2015; Puškárová et al., 2016; Elhagrassy, 2018; Liu et al., 2018b; Stratigaki et al., 2024).

The National Museum “Casa Histórica de la Independencia” (CHM), located in San Miguel de Tucumán, Argentina, stands as a vivid example of how heritage and identity intersect. Renowned as the site where the independence of the United Provinces of the Río de la Plata was declared on July 9, 1816—a date later established as Argentine Independence Day—the CHM is among the most visited historical museums in the country (Chatruc, 2021). In addition to its architectural significance, the museum preserves an extensive collection of historical furniture, documents, a large library, and a photographic archive. Among its most valuable items is *Provincia de Tucumán* by Arsenio Granillo, a 213-page volume commissioned by Governor Federico Helguera. This rare publication (Granillo, 1872), which includes original albumen prints depicting the city and its sugar mills of photographer Angel Paganelli, features the only known photograph of the museum’s original facade — a crucial visual reference during the building’s restoration in 1943.

The objective of this study is to provide the first comprehensive microbiological characterization of the CHM collections, using them as models to investigate the role of cultivable bacteria in the biodeterioration of heritage materials. We aim to identify and classify viable bacterial strains colonizing historical artifacts and architectural elements, determine the prevalence of specific taxa in relation to substrate types and microenvironmental conditions, and assess the functional potential of these microorganisms—particularly their capabilities for biodegradation and resistance to environmental stressors. Additionally, this study seeks to evaluate the potential biohazards posed by microbial communities in museum environments, contributing to the development of more effective preventive conservation strategies. To achieve these goals, we employed an integrative approach combining scanning electron microscopy (SEM) for visualizing microbial colonization, culture-dependent methods for bacterial isolation, MALDI-TOF mass spectrometry for rapid taxonomic identification, and whole-genome sequencing (WGS) for functional profiling.

## 2 Materials and methods

### 2.1 Brief description of the Casa Histórica museum

The CHM is located in San Miguel de Tucumán, the capital of Tucumán Province, Argentina at a pedestrian zone with significant foot traffic.^1^ The Casa Historica is a preserved colonial-style building and functions as a museum, offering guided tours and exhibitions related to Argentina’s independence and the revolutionary era. The museum features four courtyards, a cistern, and native trees from the region. It currently holds over 700 items in its collection including the original Act of the Declaration of Independence and various other pieces such as period furniture, weapons, personal items, dishware, religious artifacts, paintings, portraits, coins, and commemorative plaques and medals from the 18th and 19th centuries. The museum’s library contains classic texts, recent historiographical debates, museum studies, conservation, restoration, literature, journals and early provincial newspapers. Moreover, the Museum conserves an extensive book library and a photographic archive. Among the highlights is Provincia de Tucumán by Arsenio Granillo, a 213-page book (Granillo, 1872) commissioned by Governor Federico Helguera, features descriptive articles and news about Tucumán, accompanied by original albumen print photographs by Angel Paganelli, depicting the city and its sugar mills.

### 2.2 Selection of the artifacts for the sampling

Before sampling, an extensive survey and photographic documentation were conducted to capture the condition of cultural heritage items affected by different types of deterioration. This preliminary assessment informed the selection of specific sampling locations within the building and on key artifacts of cultural significance.

**Figure 1:**
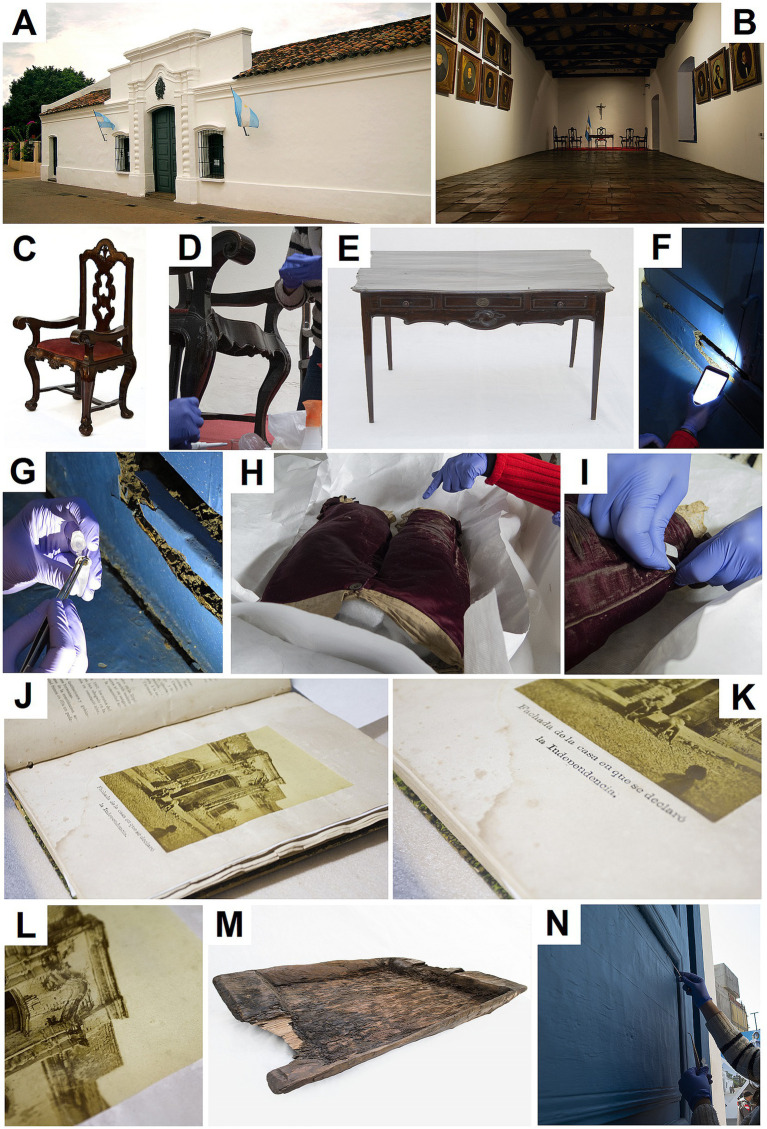
**(A)** Exterior facade of the museum. *B. interior* of the “Salón de la Jura,” **(B)** a historic room central to the declaration of Argentine independence. **(C–E)** Furniture from the “Salón de la Jura”: **(C)** colonial chair; **(D)** detail of chair leg and surface during sampling; **(E)** table sampled for microbial analysis. **(F,G)** window from the “Salón de la Jura”: **(F)** overview of the window structure; **(G)** close-up of the deteriorated frame during sampling. **(H,I)** Garment belonging to Juan Bautista Alberdi: **(H)** general view of preserved trousers; **(I)** detail of fiber structure showing signs of microbial alteration. **(J–L)** Nineteenth-century albumen print photo-book: **(J)** open view of interior; **(K)** detail showing biological staining and foxing; **(L)** close-up of albumen photograph. **(M)** Historic wooden washing trough recovered from the museum’s collection. **(N)** Main entrance door of the museum, sampled at the lower panel. Ph: Cecilia Gallardo y Alejandro Torres.

The initial phase of sampling took place in August 2019. Samples were taken from the museum’s exterior, including facade elements like the front door and painted walls (Figure 1A), which are subjected to annual repainting, weathering, and urban pollution. Within the museum, samples were taken from the window and furniture in the “Salón de la Jura” (Figure 1B). Additional historical artifacts that required preservation evaluations were also sampled (Table 1 and Figure 1).

**Table 1:** Taxonomic classification, morphological description, and microscopy-based traits of bacterial strains isolated from the Casa Histórica de la Independencia Museum (CHM).

ID	Taxonomical identification by VITEK	Source	Macroscopical characterization		Microscopical characterization	
			Colour	Surface and texture	Morphology	Gram
CH - 001	*Micrococcus luteus*	SJ Chair (textile)	Intense-yellow	Smooth and creamy	Coccoid	(+)
CH - 002	*Staphylococcus equorum*	SJ Chair (textile)	Pale-yellow	Smooth and creamy	Coccoid	(+)
CH - 003	*Bacillus simplex*	SJ Chair (textile)	Cream-colored	Smooth and creamy	Bacillus	(+)
CH - 004	*Bacillus licheniformis*	SJ Chair (textile)	Cream-colored	Smooth and creamy	Bacillus	(+)
CH - 005	*Micrococcus* sp.	SJ Chair (wood)	Orange	Smooth and creamy	Coccoid	(+)
CH - 006	*Kocuria rosea*	SJ Chair (wood)	Orange	Smooth and creamy	Coccoid	(+)
CH - 007	*Unidentified*	SJ Chair (wood)	Pale-yellow	Smooth and creamy		
CH - 008	*Kocuria rosea*	SJ Table (wood)	Orange	Smooth and creamy	Coccoid	(+)
CH - 009	*Micrococcus luteus*	SJ Table (wood)	Intense-yellow	Smooth and creamy	Coccoid	(+)
CH - 010	*Bacillus altitudinis/pumilus*	SJ Table (wood)	Cream-colored	Rough and mucoid	Bacillus	(+)
CH - 011	*Staphylococcus equorum*	Washing trough (wood)	Pale-yellow	Smooth and creamy	Coccoid	(+)
CH - 012	*Bacillus* sp.	Washing trough (wood)	Cream-colored	Smooth and creamy	Bacillus	(+)
CH - 013	*Actinomyces odontolyticus*	Alberdi’s Suit (silk and lace)	White	Dry and hard	Mycelium	(+)
CH - 014	*Micrococcus luteus*	SJ Window (blue painted wood)	Intense-yellow	Smooth and creamy	Coccoid	(+)
CH - 015	*Microbacterium aurum*	SJ Window (blue painted wood)	Yellow	Smooth and creamy	Bacillus	(+)
CH - 016	Unidentified	SJ Window (blue painted wood)	Yellow	Smooth and creamy		
CH - 017	*Microbacterium aurum*	Entrance door (blue painted wood)	Yellow	Smooth and creamy	Bacillus	(+)
CH - 018	*Bacillus altitudinis/pumilus*	Entrance door (blue painted wood)	Pale-yellow	Smooth and creamy	Bacillus	(+)
CH - 019	*Clostridium subterminale*	Entrance door (blue painted wood)	Yellow	Smooth and creamy	Bacillus	(+)
CH - 020	*Micrococcus luteus*	Entrance door (blue painted wood)	Intense-yellow	Smooth and creamy	Coccoid	(+)
CH - 021	*Corynebacterium pseudodiphtheriticum*	Entrance door (blue painted wood)	Orange	Smooth and creamy	Bacillus	(+)
CH - 022	Unidentified	Entrance door (blue painted wood)	Yellow	Smooth and creamy		
CH - 023	*Kocuria rosea*	Entrance door (blue painted wood)	Orange	Smooth and creamy	Coccoid	(+)
CH - 024	*Corynebacterium pseudodiphtheriticum*	Exterior walls (white painted bricks)	Intense-orange	Smooth and creamy	Bacillus	(+)
CH - 025	*Kocuria rosea*	Exterior walls (white painted bricks)	Orange	Smooth and creamy	Coccoid	(+)
CH - 026	*Turicella otitidis*	Exterior walls (white painted bricks)	Yellow	Smooth and creamy	Bacillus	(+)
CH - 027	*Bacillus megaterium*	Exterior walls (white painted bricks)	Cream-colored	Smooth and hard	Bacillus	(+)
CH - 028	*Kocuria rosea*	Exterior walls (white painted bricks)	Orange	Smooth and creamy	Coccoid	(+)
CH - 029	*Bacillus altitudinis/pumilus*	GB Albumen print	Cream-colored	Smooth and creamy	Bacillus	(+)
CH - 030	Unidentified	GB Albumen print	Intense-orange	Smooth and mucoid	Coccoid	(+)
CH - 031	*Bacillus galactosidilyticus*	GB Albumen print	Cream-colored	Smooth and creamy	Bacillus	(+)
CH - 032	*Pseudomonas* sp.	GB Albumen print	Cream-colored	Smooth and creamy	Rod-shaped	(−)
CH - 033	*Pseudomonas* sp.	GB Albumen print	Cream-colored	Smooth and creamy	Rod-shaped	(−)
CH - 034	*Pseudomonas* sp.	GB Albumen print	Cream-colored	Smooth and creamy	Rod-shaped	(−)
CH - 035	*Kocuria rosea*	GB Albumen print	Pale-yellow	Smooth and creamy	Coccoid	(+)
CH - 036	*Streptomyces griseus*	GB Albumen print	White	Dry and hard	Mycelium	(+)
CH - 037	Unidentified	GB Albumen print	Cream-colored	Smooth and creamy	Bacillus	(+)
CH - 038	*Kocuria rosea*	GB Albumen print	Intense-orange	Smooth and creamy	Coccoid	(+)
CH - 039	*Bacillus galactosidilyticus*	GB Albumen print	Cream-colored	Smooth and mucoid	Bacillus	(+)
CH - 040	*Pseudomonas* sp.	GB Albumen print	Pale-yellow	Smooth and mucoid	Bacillus	(+)
CH - 041	*Bacillus thermoamylovorans*	GB Albumen print	Cream-colored	Smooth and creamy	Bacillus	(+)
CH - 042	*Streptomyces* sp.	GB Albumen print	Pale-cream	Smooth and hard	Mycelium	(+)
CH - 043	*Micrococcus luteus*	GB Albumen print	Intense-yellow	Smooth and creamy	Coccoid	(+)
CH - 044	Unidentified	GB Albumen print	Pale-yellow	Smooth and mucoid		(+)
CH - 045	*Bacillus cereus* group	GB Albumen print	Cream-colored	Smooth and creamy	Bacillus	(+)
CH - 046	*Staphylococcus epidermis*	GB Albumen print	Cream-colored	Smooth and dry	Coccoid	(+)
CH - 047	*Micrococcus luteus*	GB Albumen print	Intense-yellow	Smooth and creamy	Coccoid	(+)
CH - 048	Unidentified	GB Albumen print	Orange	Smooth and mucoid	Coccoid	(+)
CH - 049	*Pseudomonas* sp.	GB paper sheet	Cream-colored	Smooth and creamy	Rod-shaped	(−)

This table presents 49 cultivable bacterial isolates recovered from heritage artifacts and architectural elements of the CHM. For each strain, the VITEK-based taxonomic assignment is listed. The source column specifies the sampled object (e.g., photograph, textile, wood, and wall). Macromorphological traits, including colony color, surface appearance, and texture, were recorded after 24–48 h growth on LB agar. Micromorphological observations, including Gram reaction and cell shape, were determined via light microscopy. Strains are curated in the CeBAC collection under the specified isolate codes.

The Salón de la Jura or Hall of the Oath is a central room within the CHM (Figure 1B). Samples for our study were collected from one chair where congressmen sat and from the table where the Declaration of Independence was signed; this furniture remains in its original state, dating back to 1810. The wooden frame from the armrest and backrest and the velvet-covered seat of one of the chairs were sampled to assess the bacteria present on these distinct materials (Figures 1C,D). The table’s wooden surface was swabbed in multiple locations, especially in the areas less exposed to regular cleaning (Figure 1E). Additionally, we took samples from biodeteriored zones of one of the windows of the room, subjected to infestation by woodworm eggs (Figures 1F,G).

Juan Bautista Alberdi (1810–1884) was a distinguished statesman, diplomat, and writer who played a pivotal role in shaping Argentina’s political landscape. The Museum holds a captivating collection of artifacts attributed to Alberdi, including a childhood suit made from natural red silk which was sampled (Figures 1H,I).

The second phase of sampling in December 2021 targeted a key artifact from the CHM: the 1872 book *Provincia de Tucumán* authored by Dr. Arsenio Granillo (Figures 1J–L). Stored in a metallic safe case to preserve its condition, this book has survived with minimal light exposure, thermal fluctuations, and limited handling. Samples were taken from both the albumen print and the paper support.

### 2.3 Sampling procedures for direct material observation and bacterial isolation

To conduct the sampling, a non-invasive approach was employed, using precision tools to extract material samples without compromising the structural integrity of the pieces. Micro-sampling techniques, supported by visual documentation and environmental controls, were used to assess the physical condition of the albumen prints and the rest of the elements identify potential areas of degradation, such as yellowing, flaking, or damage from previous handling.

For the direct observation of microorganisms on heritage objects, freshly-open 3 M™ adhesive tape was used (Michaelsen et al., 2009). Strips of 2–3 cm were cut in advance using sterile scissors under aseptic conditions and sterilized by exposure to germicidal UV-C radiation for 15 min. Subsequently, the strips were gently adhered to the different surfaces and then transferred to 2 mL tubes containing Karnovsky’s fixative solution (2.66% w/v paraformaldehyde and 1.66% w/v glutaraldehyde in 0.1 M phosphate buffer, pH 7.2), for transportation to the lab. The tape sampling method was not used for the book of Granillo, to avoid any undesired consequences over its conservation.

For isolation of individual bacterial strains, sample collection was performed with dry and sterilized cotton swabs. The swab was passed over the entire area of visibly damaged material; each swab covered approximately a 10 cm^2^ area to standardize sample collection. Immediately after sampling, the swabs were used to inoculate plates with Luria-Bertani (LB) agar media. In most cases, selective media were used: parallel LB agar plates were supplemented with nalidixic acid (10 μg mL^−1^) and cycloheximide (10 μg mL^−1^) to inhibit the growth of Gram-negative bacteria and fungi, respectively (Rasuk et al., 2017). However, in the case of the albumen book, only non-selective LB medium was used to favor the recovery of a broader spectrum of microbial taxa, including both Gram-positive and Gram-negative bacteria. Parallel agar plates were supplemented with nalidixic acid (10 μg mL^-1^) and cycloheximide (10 μg mL^-1^) to inhibit the growth of Gram-negative bacteria and fungi, respectively (Rasuk et al., 2017). This non-invasive method is considered essential for the study of collections in museum environments (Michaelsen et al., 2009; Piñar et al., 2015). Once the colonies appeared, they were collected with a sterile loop for purification and subsequent streaking allows generating axenic cultures. We stored the cells in glycerol 20% at −20°C. Subcultures were performed in LB broth for morphological and molecular identification analysis. From the pure cultures of the bacterial isolates, macroscopic practices were carried out to determine the structure of the colonies and their pigmentation, and microscopic practices (optical and electronic) of individual cells to establish shapes, sizes, and spatial arrangement. Additionally, the Gram reactions of all isolates were recorded.

All isolated strains in this work are kept and curated in the CeBAC collection (Cepario de Bacterias Ambientales del CIME), Catalogue Urban Microbiome Strains, under the corresponding codes indicated in Table 1.

### 2.4 Scanning electron microscopy (SEM)

For SEM studies, the adhesive tapes or pure axenic cultures of isolated strains were fixed in a mixture of aldehydes (Karnovsky fixative) overnight at 4°C, and were prepared following the processing method already previously described in Alonso-Reyes et al. (2021). During post-fixation, samples were washed with 0.1 M phosphate buffer twice and dehydrated in a graded concentration series of ethanol (50, 70, 90, and 100%), following acetone 100%. This was followed by critical point drying (Denton Vacuum model DCP-1). The samples were then mounted on aluminum supports and subjected to gold sputtering (JEOL model JFC-1100) and examined using a Zeiss Supra 55VP scanning electron microscope (Carl Zeiss NTS GmbH, Germany), in the Electron Microscopy Core Facility (CIME-CONICET-UNT).

### 2.5 Identification of microbial strains by MALDI-TOF MS (VITEK)

MALDI-TOF analysis was performed with a bioMérieux VITEK MALDI-TOF mass spectrometer (bioMérieux, France), and the spectra were compared to the VITEK MS SARAMIS database for research use only (RUO), version 4.09, using the SuperSpectra algorithm (referred to here as MALDI-TOF MS). For MALDI-TOF MS analysis, a colony from a pure plate with 24 h growth was applied to a slide and covered with 1 μL of matrix solution (*α*-cyano-4-hydroxycinnamic acid) and dried completely before analysis. The identification was considered definitive when the probability provided was >70%. This service was provided by Laboratorios CIBIC in Rosario, Argentina.

### 2.6 DNA extraction

Eight representative bacterial genomes were selected for DNA sequencing (Table 2) to represent the ecological and substrate diversity of the CHM samples, including three isolates from outdoor surfaces, two from the indoor furniture surfaces of the well-preserved Salón de la Jura, and three strains from a uniquely preserved photographic artifact. The selection was guided by phenotypic variability, distinct cultural origin, and presumed relevance to biodeterioration processes, as determined by preliminary MALDI-TOF MS identification and morphological traits. The DNA was obtained using a commercial extraction kit (DNA Puriprep B-kit for bacteria, Inbio Highway, Argentina); following the manufacturer’s instructions. For Gram-positive bacteria, the pellet was suspended in 162 μL of BRB buffer and 18 μL of lysozyme, and then incubated for 30 min at 37°C. For gram-negative bacteria, the use of lysozyme was omitted. Next, 200 μL of BT buffer and 20 ul of proteinase K were added to each sample. It was incubated for 30 min at 56°C. After, RNaseA (10 mg/mL), was added and incubated for 30 min at 37°C. Then the lysis buffer (200 μL) was added, vortex pulsed for 10 s and the homogenate was incubated for 10 min at 56°C. Then, 200 μL of 100% ethanol was added to each sample, shaken by inversion, and transferred to a microcolumn. It was centrifuged for 1 min at 12,000 g, and the filtrate was separated. Then, two column washes were performed each with 500 μL of Blav1 and Blav2 buffer, respectively. Finally, the column was dried by centrifuging for 3 min at 12,000 g. DNA elution was carried out with 60 μL of pH 9 elution buffer, equilibrated at 70°C. DNA concentration and quality were measured using a μDrop plate (Thermo Scientific).

**Table 2:** Genomic sequencing, assembly statistics, and taxonomic identification of selected bacterial strains isolated from the CHM.

Strain code	CH-041	CH-031	CH-036	CH-007	CH-015	CH-021	CH-019	CH-026
Isolation place	Albumen picture	Albumen picture	Albumen picture	SJ Chair (wood)	SJ Window	Main door	Main door	Outside wall
Raw data (Gb)	1.4	1.5	1.6	2.3	2.1	2.2	1.8	2.2
Length after assembly (bp)	4,277,215	3,925,509	7,608,646	3,319,459	3,250,763	3,907,984	3,187,239	3,685,277
Contigs (≥200 nt)	113	33	79	20	42	80	28	56
CDS	4,067	3,925	6,773	3,143	3,063	3,584	3,223	3,416
Annotated genes	3,664	3,737	6,577	2,887	2,766	3,208	2,988	2,909
Taxonomy with Vitek	*Bacillus thermoamylovorans*	*Bacillus galactosidilyticus*	*Streptomyces griseus*	*Unidentified*	*Microbacterium aurum*	*Corynebacterium pseudodiphtheriticum*	*Clostridium subterminale*	*Turicella otitidis*
Taxonomy with 16S/gyrB	*Caldibacillus thermoamylovorans (gyrB)*	*Oceanobacillus* sp. *(gyrB)*	*Streptomyces globisporus (16S)*	*Microbacterium imperiale (16S)*	*Microbacterium aurum* (16S)	*Kocuria rosea* (16S)	*Bhargavaea ginsengi* (16S)	*Pseudoclavibacter* sp. (16S)
Taxonomy with ANI	*Caldibacillus thermoamylovorans*	*Oceanobacillus kimchii*	*Streptomyces rubiginosohelvolus*	*Microbacterium* sp.	*Microbacterium aurum*	*Kocuria* sp.	*Bhargavaea massiliensis*	*Pseudoclavibacter* sp.
Closest match in NCBI genomes	*Caldibacillus thermoamylovorans* MSL 185.1 *Spacecraft cleanroom* ASM2371276v1 (97.93%)	*Oceanobacillus kimchii* p3-SID1558 *Human back skin* ASM2514966v1 (99.47%)	*Streptomyces* sp. f150 *Soil* ASM255143v1 (98.48%)	*Microbacterium arborescens* *Leaves of Ricinus comunis* ASM333966v1 (87.82%)	*Microbacterium* sp. DE14.009 *Geothermal springs* ASM1476380v1 (97.98%)	*Kocuria* sp. *Duodenal mucosa of celiac disease patient* ASM148375v1 (98.71%)	*Bhargavaea massiliensis* *Marseille-Q1000 human urine* GCA_902825275.1 (99.32%)	*Pseudoclavibacter helvolus PS02339* *Danish slaughterhouse wall* ASM3001191v1 (87.11%)

This table summarizes key genomic features of eight representative bacterial isolates selected from distinct environments within the Casa Histórica de la Independencia Museum (CHM), including the albumen print photograph, museum furniture, and exterior surfaces. For each strain, the table includes: (i) the isolation source (artifact or location), (ii) total amount of raw sequencing data generated (in gigabases), (iii) genome assembly length (in base pairs), (iv) number of assembled contigs ≥200 nt, and (v) predicted number of protein-coding sequences (CDS). Whole genome sequencing was performed using Illumina NovaSeq PE150 technology. Assemblies were generated with SPAdes v3.15.4, and functional annotation was conducted using the Arche pipeline. Taxonomic identifications were refined using VITEK-MS, 16S and gyrB gene markers, and ANI-based comparisons against reference genomes in NCBI.

### 2.7 Genome sequencing, assembly, and analysis

Whole genome sequencing service was performed by Novogene UK services^2^ using the Illumina Novaseq platform with pair-end 150 strategy. Quality control of the reads was also performed by Novogene. The Illumina reads were assembled using SPAdes assembler v. 3.15.4 (Bankevich et al., 2012), with—careful and—cov-cutoff auto options. The resulting eight assemblies were uploaded to the NCBI database under the Bioproject accession number PRJNA1233209, and are also available at Zenodo.^3^ The sequencing process and quality control yielded approximately 100 million reads and 15 Gb of data, with an average of 12 million reads and 1.88 Gb per genome. Assembly resulted in fewer than 115 contigs for all strains, except for the CH-041 strain, which had 200 contigs. The functional annotation was performed using Arche v. 1.0 (Alonso-Reyes and Albarracín, 2025) pipeline with—kegg option, and the resultant KEGG identifiers (KO) were mapped through the KEGG mapper reconstruct tool.^4^ The Arche pipeline, employing GeneMarkS-2 for gene prediction, achieved high annotation rates for protein-coding sequences (CDS). The lowest annotation rate was observed in CH-026, with ~85% of CDS annotated, while CH-036 reached approximately 97% annotation. The genomic potential for the synthesis of secondary metabolites was elucidated through antiSMASH v. 7.1.0 (Blin et al., 2023). To estimate the taxonomic identity of each strain at genus level, 16 s-rRNA and DNA gyrase B genes were retrieved from the genomes using barrnap and blast tools, and then blasted against the NCBI NR database. For complete taxa identification, average nucleotide identity (ANI) analysis was performed using pyani (Pritchard et al., 2016), against genus-specific genomes deposited in NCBI database. No statistical analyses were applied in this study, as the focus was on descriptive microbial characterization using microscopy, culture-based isolation, MALDI-TOF identification, and genomic analyses.

## 3 Results

### 3.1 SEM analysis of microbial communities on heritage objects and facade surfaces

**Figure 2:**
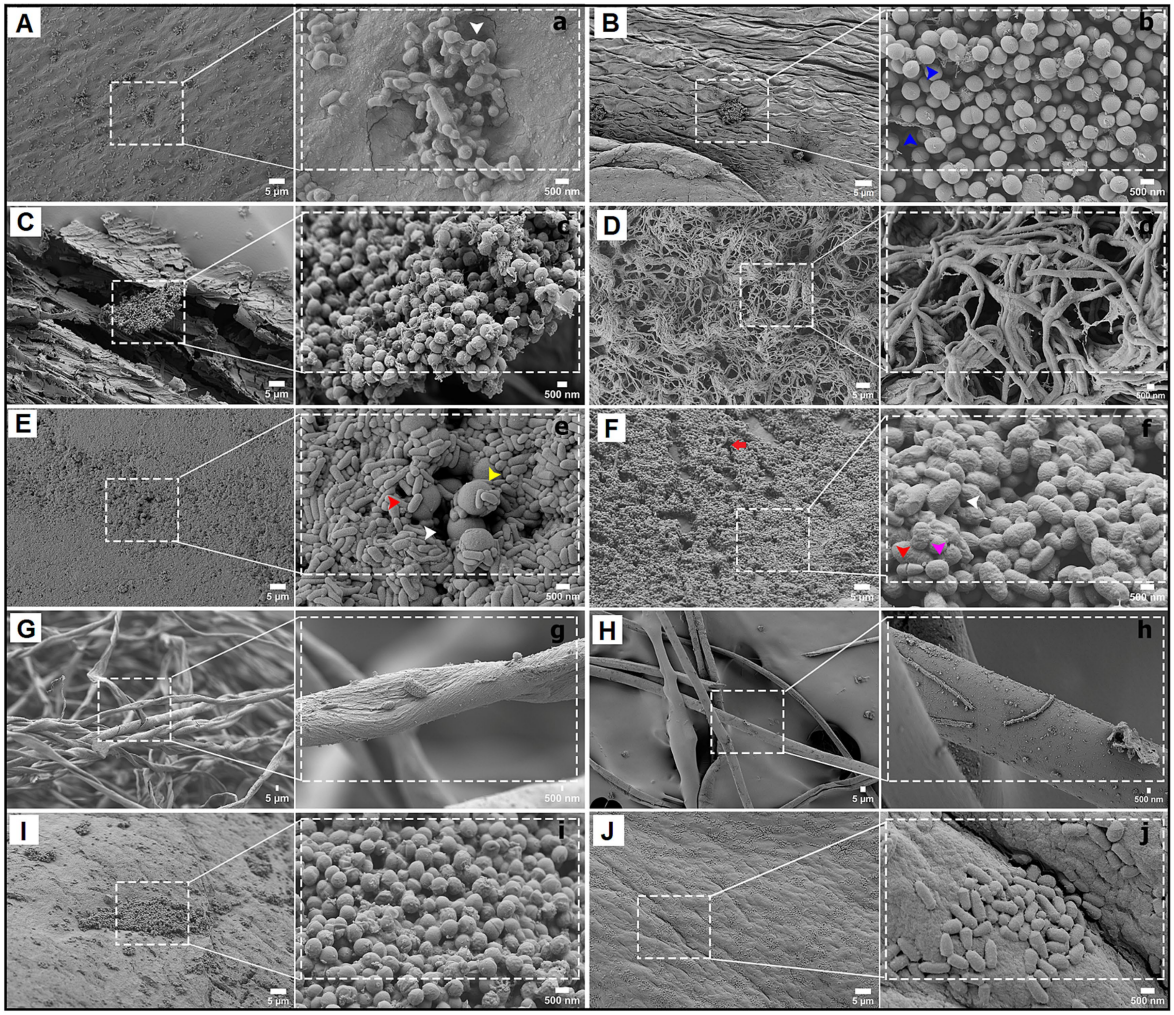
Scanning electron microscopy (SEM) of biofilms detected on heritage surfaces from the Casa Histórica de la Independencia Museum (CHM). Each panel displays colonized materials sampled from key museum artifacts. Left subpanels show wide field images (5 μm scale), while right subpanels present higher magnifications of microbial structures (500 nm scale). **(A,a)** Chair armrest showing spindle-shaped and hazelnut-like bacterial cells embedded in an extracellular polymeric substance (EPS) matrix. **(B,b)** Table surface colonized by coccoid bacterial cells interconnected by thin filaments (blue arrowheads). **(C,c)** Washing trough showing clusters of coconut-shaped biofilm-forming bacteria within cracks of the wood. **(D,d)** Filamentous network likely corresponding to Actinobacteria (e.g., *Streptomyces*). **(E,e)** Homogeneous mat of short bacilli (red arrowhead), with sporulated forms (yellow arrowhead) embedded in EPS. **(F,f)** Multispecies biofilm on wooden surface containing cocci (pink arrowhead), bacilli (red arrowhead), and irregular morphologies (white arrowheads), with clear spatial organization. **(G,g)** Fibrous textile structure from Alberdi’s garment with bacterial filaments entangled with fabric strands. **(H,h)** Sparse colonization of textile fibers showing limited microbial structures and occasional debris. **(I,i)** Surface of a window plank with compact coccoid cells embedded in minimal EPS. **(J,j)** Main entrance door showing a consolidated biofilm of cocci and bacilli within surface depressions.

Biofilms on heritage surfaces including furniture, wooden objects (chair, table, window and washing trough) and textile garments were analyzed using scanning electron microscopy (Figure 2). In all cases, the adhesive tape sampling method proved to be a useful, simple, and non-destructive technique for collecting microbial communities for immediate imaging analysis. The surfaces of objects within and outside the museum were highly colonized with microbes forming well-structured biofilms.

Most observed biofilms exhibited three-dimensional cellular organization and were surrounded by substantial amounts of extracellular material. For instance, the wooden chair showed spindle or hazelnut-shaped bacterial cells embedded in an abundant exopolysaccharide (EPS) matrix (Figure 2A). The wooden table displayed a homogeneous bacterial biofilm with coccoid units connected by thin, short filaments (Figure 2B). In the wooden washing trough, compact coconut-shaped biofilms were observed attached to wooden rests (Figure 2C), while forming an interconnected by extracellular filaments or vesicles. Other areas revealed a filamentous bacterial mantle (Figure 2F) likely belonging to the genus *Streptomyces* or related Actinobacteria. On garments, microbial filaments were much scarce (Figures 2G,H); they were adhered tightly to deteriorated fibers of the clothing, with fibrous materials and deposits visible at higher magnifications. In this sample, diverse fungal spores were also found. In the window sample, we observed a biofilm formed by heterogeneous microbial community dominated by large cocci and compacted bacilli, with minimal extracellular material facilitating cell interactions (Figure 2A).

The facade also showed high colonization of the surfaces by biofilms; the entrance door exhibited a thick biofilm structure composed of cocci, dividing bacilli, larger rough-surfaced bacilli, and filamentous bacteria, all forming strongly interconnected aggregates (Figure 2J). The exterior walls demonstrated consolidated coccoid biofilms held together by EPS, likely offering protection against environmental factors, while other regions showed sparse adherence of bacilli in simple distributions.

### 3.2 Isolation and VITEK-MS-based identification

**Figure 3:**
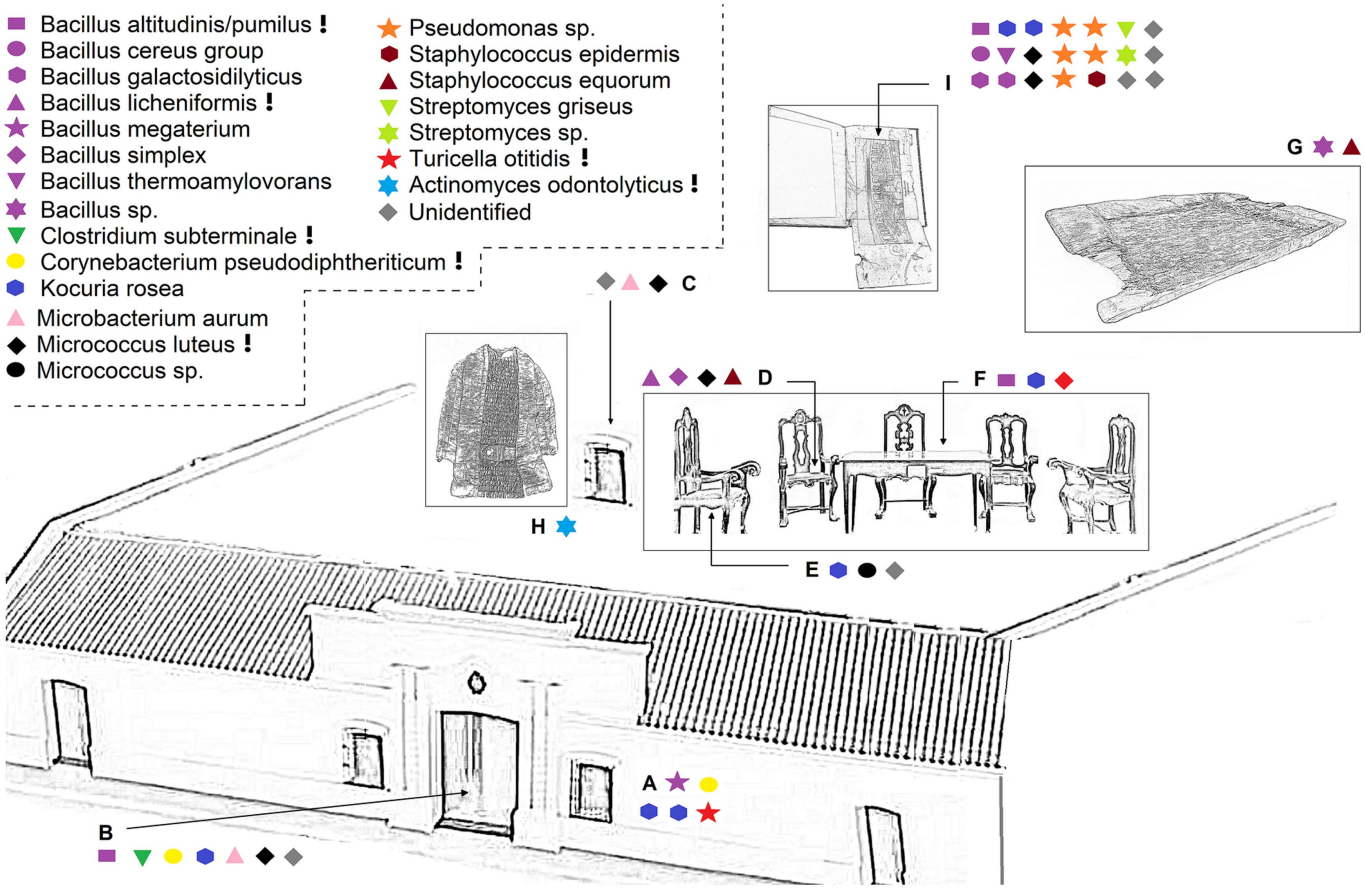
Illustration of the Casa Histórica de la Independencia Museum (CHM) and sampling sites showing the distribution of bacterial taxa identified by VITEK-MS. The schematic highlights the artifacts and surfaces sampled throughout the museum, with corresponding microbial taxa indicated by colored symbols. Shapes and colors represent distinct bacterial species or genera. Each symbol corresponds to a taxonomic group (see legend, upper left). Locations sampled include: **(A)** facade wall, **(B)** main door, **(C)** window frame **(D–F)**. Furniture from the Salón de la Jura (chairs and table) **(G)**. Wooden washing trough **(H)**. Alberdi’s childhood garment **(I)**. Albumen print book (photograph and paper surface). Symbols marked with an exclamation mark (!) indicate species with reported pathogenicity to humans or plants, based on literature references (e.g., *Staphylococcus epidermidis*, *Bacillus licheniformis*, *Turicella otitidis*, etc.). Gray diamonds denote unidentified isolates. All strain identifications are detailed in Table 1.

**Figure 4:**
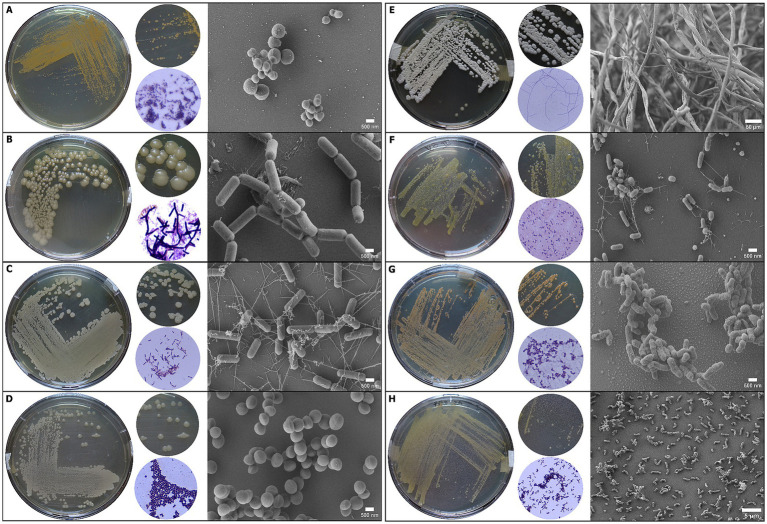
Morphological characterization of selected bacterial strains isolated from the CHM using colony morphology, Gram staining, and scanning electron microscopy (SEM). Each row **(A–H)** corresponds to an individual bacterial strain: **(A)** CH-005 (*Micrococcus* sp.), **(B)** CH-003 (*Bacillus* sp.), **(C)** CH-010 (*Bacillus* sp.), **(D)** CH-011 (*Staphylococcus* sp.), **(E)** CH-013 (*Streptomyces* sp.), **(F)** CH-015 (*Microbacterium aurum*), **(G)** CH-021 (*Kocuria* sp.), **(H)** CH-026 (*Turicella otitidis*). From left to right, each panel shows: (i) culture plate image on LB agar with colony pigmentation and texture; (ii) enlarged view of colony morphology; (iii) Gram-stained smear observed under light microscopy, indicating cell wall characteristics and arrangements; (iv) SEM micrograph showing fine-scale cell morphology and surface features. All SEM images were acquired at 500 nm or 5 μm scale depending on cellular dimensions. The combination of macroscopic, microscopic, and ultrastructural views allows accurate differentiation of morphotypes, aiding taxonomic confirmation and highlighting structural diversity among strains colonizing museum artifacts.

A total of 49 bacterial strains were isolated from various samples (Table 1 and Figures 3, 4), of which 12 were associated with the museum’s exterior surfaces and 37 with interior environments. Each sampled niche exhibited a distinct profile of bacterial taxa, reflecting the contrasting conditions of outdoor and indoor environments. Moreover, each artifact sampled from within the Casa Histórica de la Independencia can be regarded as an isolated microbial island, shaped by its unique environmental conditions, material composition, age, usage and historical context. The microbial communities inhabiting these artifacts form a complex and dynamic microecosystem, where each niche harbors a distinctive collection of bacteria specifically adapted to its substrate (Figure 3). Gram-positive bacteria dominated the isolates, as expected given the use of selective isolation media supplemented with antibiotics to exclude fungi and gram-negative bacteria. The most prolific source of isolates was the albumen photograph, where no inhibitors were used. Here 21 distinct strains were recovered, predominantly from the genera *Bacillus, Oceanobacillus, and Streptomyces*. In contrast, the Alberdi’s garment gave a unique isolate: *Actinomyces odontolyticus*. Morphological characteristics of each strain (texture, color, spore production) were recorded to differentiate isolates (Table 1).

Four strains were isolated from the textile upholstery of a chair (*Micrococcus luteus, Staphylococcus equorum, Bacillus licheniformis and Bacillus simplex*) while three strains were found on the wooden frame of the same chair (*Micrococcus* sp., *Kocuria* sp. and one unidentified strain). Additional 15 isolates were recovered from other wooden elements, including the table (*Kocuria rosea, Micrococcus luteus, Bacillus altitudinis/pumilus*), the window frame (*Micrococcus luteus, Microbacterium aurum* and one unidentified strain), the museum’s entrance door (*Microbacterium aurum*, *Bacillus altitudinis/pumilus*, *Clostridium subterminale*, *Micrococcus luteus*, *Corynebacterium pseudodiphtheriticum, Kocuria rosea* and one unidentified strain) and a washing trough (*Staphylococcus equorum* and *Bacillus* sp.) where meat of slaughter animals was prepared. Finally, five strains were isolated from the front wall of the building made of white painted bricks (*Corynebacterium pseudodiphtheriticum, Kocuria rosea, Turicella otitidis, Bacillus megaterium*).

### 3.3 Genome-based taxonomical identification

Although VITEK MALDI-TOF MS was used for preliminary identification, higher-resolution taxonomic resolution was achieved using 16S rRNA and *gyrB* gene markers and refined through ANI analyses, which provided species-level assignment where reference genomes were available. The gyrB gene was used when the 16S-rRNA gene was incomplete or truncated. ANI analyses were performed using genus-specific genomes from the NCBI database for more precise identification (Table 2).

VITEK identification results for most strains differed from those obtained through MGs. Only strains CH-041 and CH-015 maintained the same species classification by both methods. CH-036 was identified as belonging to the genus *Streptomyces* by VITEK, MGs, and ANI, but the species differed between methods. VITEK accurately classified CH-031 at the family level (Bacillaceae), but MGs identified it at the genus level as *Bacillus*, whereas ANI suggested *Oceanobacillus*. CH-021 and CH-026 only matched at the class level (Actinomycetes) by both methods, while CH-019 matched at the phylum level (Bacillota). CH-007 could not be classified by VITEK but was identified as *Microbacterium* by MGs and ANI. Significant differences were evident in species-level classifications; MGs and ANI agreed on the species for CH-041 (*Caldibacillus thermoamylovorans*) and CH-015 (*Microbacterium aurum*). In CH-031, where MGs did not yield a species classification, ANI clustered it with *Oceanobacillus kimchi* due to >95% similarity to a reference genome in the NCBI database (Richter and Rosselló-Móra, 2009). Notably, CH-007, and CH-026 did not align with any reference genome above the 95% ANI threshold, suggesting they might belong to novel species.

Pairwise comparisons between the CHM genomes and publicly available genomes revealed their closest phylogenetic matches (Table 2). Most strains exhibited >97% sequence similarity; however, CH-026 and CH-007 showed lower similarities of 87.11 and 87.82%, respectively. Notably, three strains — *Oceanobacillus kimchi* CH-031, *Kocuria* sp. CH-021, and *Bhargavaea massiliensis* CH-019 — showed high-confidence matches to species commonly associated with the human microbiome (Table 2). The G + C content varied largely between genomes, ranging from ~35% in CH-031 to ~72% in CH-021.

### 3.4 Functional annotation of genomes: insights into biodeterioration and environmental resilience

**Figure 5:**
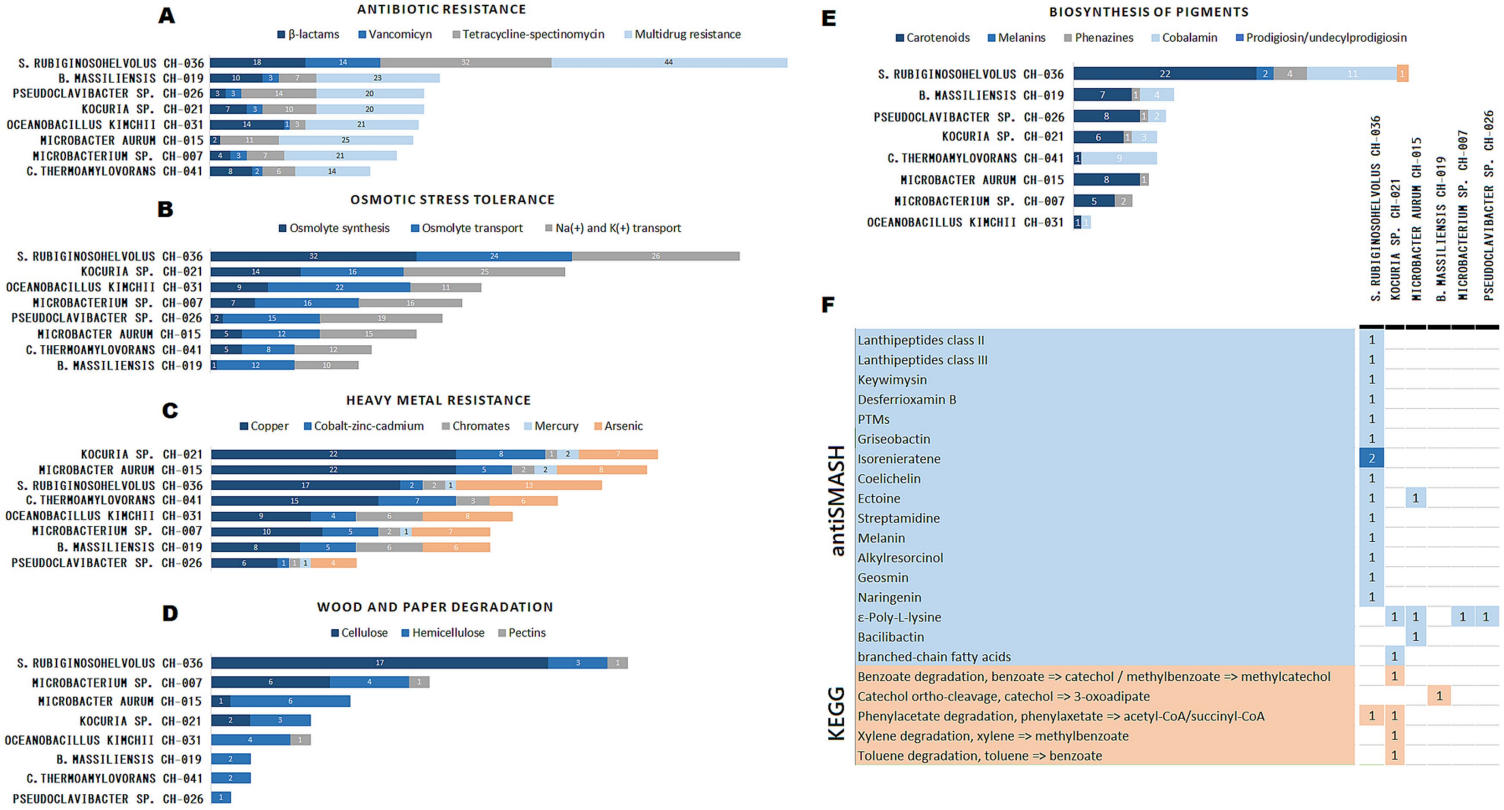
Genomic functional potential of eight sequenced strains from the CHM, based on KEGG and antiSMASH annotations. **(A-E)**. Bar plots showing the number of genes annotated in five functional categories: **(A)** Resistance to antibiotics (*β*-lactams, vancomycin, tetracycline/spectinomycin, multidrug resistance). **(B)** Osmotic stress tolerance (osmolyte synthesis/transport, cation transporters). **(C)** Toxic metal resistance (copper, zinc/cadmium, chromates, mercury, arsenic). **(D)** Wood and paper degradation (cellulose, hemicellulose, pectins). **(E)** Pigment biosynthesis (carotenoids, melanins, phenazines, cobalamin, prodigiosin/undecylprodigiosin). **(F)** Presence of biosynthetic gene clusters (BGCs) for secondary metabolite synthesis (blue) and xenobiotic degradation pathways (orange), inferred through antiSMASH and KEGG, respectively. Only six of the eight strains are shown in **(F)**, as CH-031 (*Oceanobacillus kimchii*) and CH-041 (*Caldibacillus thermoamylovorans*) did not display any detectable biosynthetic gene clusters or xenobiotic degradation pathways under the criteria used by antiSMASH and KEGG. This analysis highlights CH-036 (*Streptomyces* sp.) and CH-021 (*Kocuria* sp.) as the most biosynthetically active and environmentally resilient strains, with relevance to both biodeterioration and biotechnological potential.

The functional annotation carried out using Arche led to useful information regarding the genomic potential of the strains (Figures 5A–E and Supplementary Tables S1–S15). Genes involved in resistances to antibiotics, osmotic stress, and toxic metals were reported from the annotation. Furthermore, relevant genes related to the biodeterioration of the cultural heritage were found, such as those required for the degradation of wood and paper, and the production of pigments. *Streptomyces* sp. CH-036 revealed the highest numbers of genes in all the systems mentioned before; except for toxic metal resistance (the numbers are still relatively high). Moreover, CH-036 reported more than twice as many genes related to biodeterioration (Figures 5D,E) as the rest of the strains studied. Particularly, those genes related to the degradation of cellulose and production of pigments, which are potentially harmful to paper documents, were highly abundant in CH-036. *Kocuria* sp. CH-021 strain displayed a high potential to resist both the osmotic and toxic metal stresses. A relatively elevated number of cation transporters were annotated in CH-021’s genome, and genes related to copper and chromate homeostasis were also noticeable. Genes for the degradation of cellulose and hemicellulose are also present, together with the capability to produce some pigments. On the other hand, both *Oceanobacillus* sp. CH-031 and *Caldibacillus* sp. CH-041 had a reduced biodeteriorative potential; a tiny group of genes devoted to pigment production and degradation of wood and paper were found with none of the latter related to cellulose degradation. Finally, CH-041 showed a clear toxic metal resistance profile considering the coding-sequences found in the genome.

Further functional capabilities of relevance were studied, such as the potential for the synthesis of secondary metabolites, and degradation of xenobiotics (Figure 5F). The results of the screening with antiSMASH revealed *Streptomyces* sp. CH-036 as the most diverse of the strains regarding to the hosting of biosynthetic gene clusters (BGCs) devoted to the production of secondary metabolites. Among the putative products of these BCGs are lanthipeptides, and *ε*-poly-L-lysines, both compounds with reported antimicrobial properties (Repka et al., 2017; Wang et al., 2021). Other compounds reported from CH-036 genome were desferrioxamin (Holden and Nair, 2019), and isorenieratene, which is a light-induced yellow pigment (Takano et al., 2005). On the other hand, KEGG identifiers retrieved from the annotation of *Kocuria* sp. CH-021 genome revealed several complete pathways for xenobiotic degradation, including those for the degradation of benzoate, phenylacetate, xylene, and toluene.

## 4 Discussion

This study revealed significant microbial colonization on historical artifacts and architectural surfaces, highlighting both the conservation challenges and the potential scientific value of these microbial communities. SEM analysis of historical artifacts showed extensive biofilm development in most samples. In some cases, like the Alberdi’s garment, it was observed tightly adhered microbial filaments to altered original material of the pieces, providing direct evidence of biodeterioration processes that threaten cultural heritage.

Microbial colonization in the CHM is shaped by distinct ecological niches, with differences observed between the outdoor urban microbiome, the indoor museum microbiome, and the albumen print book which remains stored in a metallic case. The exteriors, primarily represented by microbial communities on the museum’s facade, entrance door, and exterior walls, is continuously exposed to fluctuating environmental conditions such as temperature variations, UV radiation, precipitation, and air pollutants. In contrast, the interiors comprise microbial communities inhabiting historical furniture, textiles, and architectural surfaces within the controlled indoor environment of the museum. These niches are characterized by relatively stable temperature and humidity conditions, lower UV exposure, and reduced air circulation. Finally, the book endures minimal light exposure, thermal fluctuations, and limited handling which could favor specialized communities.

It is important to acknowledge that the use of LB medium in the isolation procedures likely introduced a bias toward fast-growing, copiotrophic bacteria. While this approach successfully recovered strains with potential relevance to biodeterioration processes, it inevitably captures only a fraction of the microbial diversity present in the sampled environments. The rich nutrient content of LB does not reflect the oligotrophic and substrate-specific conditions typically found on cultural heritage materials. Consequently, slow-growing and metabolically specialized microorganisms may have been underrepresented in our isolate collection. Future efforts could benefit from using selective or minimal media supplemented with heritage-relevant substrates, such as albumin, cellulose, or lignin, to recover a broader spectrum of metabolically active and ecologically relevant taxa. Nonetheless, the genomic and phenotypic insights derived from the isolates obtained here provide valuable initial data for understanding microbial contributions to biodeterioration in museum contexts. Finally, as such isolates may not fully represent the actively growing microbial community responsible for ongoing biodeterioration, their ecological relevance should be interpreted with caution and complemented by future culture-independent analyses (Liu et al., 2022).

Worth to note is that the use of multiple complementary methods for bacterial identification allowed us to assess the strengths and limitations of each approach. While VITEK MALDI-TOF MS offered rapid initial classification, its performance was constrained by the scope of the clinical-focused database, potentially leading to misclassification or low-resolution results for environmental isolates. In contrast, genome-resolved methods based on 16S rRNA and *gyrB* sequences, along with average nucleotide identity (ANI) comparisons, provided superior accuracy and confidence in taxonomic assignment, particularly for uncommon or novel strains. Therefore, for species-level resolution, ANI proved to be the most reliable method in this study. This multi-method approach also allowed us to identify potential novel species and refine our understanding of microbial diversity associated with heritage materials.

The albumen prints in the book Provincia de Tucumán represent a unique microenvironment, as even subtle microclimatic differences can shape distinct microbial communities and deterioration patterns (Meng et al., 2023). Composed primarily of denatured egg white proteins, cellulose and salts they can be a suitable substrate for microbial colonization and biodeterioration. Several strains isolated from the CHM’s photo book were classified by VITEK, MGs, and ANI as belonging to the genera *Bacillus, Oceanobacillus, Microccocus*, *Streptomyces*, *Staphylococcus* and *Kocuria* (Table 1 and Figure 3). These taxa were consistently reported as microbial contamination in historical archives and audiovisual materials (Villalba et al., 2004; Puškárová et al., 2016; Mazzoli et al., 2018; Branysova et al., 2023). They have proven amylolytic, cellulolytic, proteolytic, and/or lipolytic capacities related to biodeterioration of paper, photographs, and/or other visual material which were mainly determined by culture-dependent methods (Villalba et al., 2004; Branysova et al., 2023). The amylolytic capacities can be used for the consumption of starch from paper (used as a glue in its manufacturing) while the proteolytic activity presents a threat especially to visual materials with a gelatinous binder. Some species with cellulolytic properties pose a risk to collodion visual materials (as the main component of collodion is cellulose nitrate) while others have the ability to produce alkaline serine proteases which are harmful to albumin materials (Villalba et al., 2004; Branysova et al., 2023).

Notably, *Pseudomonas* strains were exclusively isolated from the photograph, aligning with its role as a frequent contaminant in photographic manufacturing due to its ability to metabolize gelatin and resist silver nitrate (Gadd et al., 1989; Borrego et al., 2010; Kraková et al., 2012b; Sterflinger and Piñar, 2013; Madigan et al., 2014; Grabek-Lejko et al., 2017; Mazzoli et al., 2018; Warner Marien, 2022). *Pseudomonas* spp. and *Streptomyces* spp. poses a risk not only because of its potential catalytic properties, but also because of the ability of some members to produce deteriorative pigments. For example, some *Streptomyces* spp. ruined graphic art inside some Etruscan and Roman tombs due to the production of violet pigments, while members of *Pseudomonas* produced both green (in an alkaline environment) and red (in acidic conditions) pigments during wool degradation of historical textiles (Díaz-Herráiz, 2015; Gutarowska et al., 2016; Mazzoli et al., 2018). In accordance with culture-dependent methods, genomic analysis over the strain *Streptomyces* sp. CH-036 (Table 2) revealed strong capabilities to degrade components of paper (Figure 5D). Moreover, a high number of genes for the biosynthesis of several pigments were reported (Figure 5E) including isorenieratene (Figure 5F), a light-induced pigment (Takano et al., 2005). A similar pigment-related genes were found in *Rubrobacter* strains colonizing the pink biofilms on frescoes from Georgian cathedrals, where their persistence was linked to both light exposure and metabolic adaptability (Basile et al., 2025). These findings should encourage CHM authorities to implement precautionary measures that prevent the exposure of the albumen print book to light, humidity, or warm temperatures—conditions that could facilitate microbial proliferation. In particular, humidity and water availability are widely recognized as the primary drivers of microbial growth and subsequent biodeterioration on cultural heritage surfaces. Recent evidence highlights that even subtle moisture levels can dramatically shape microbial colonization and biochemical activity, especially in porous materials and confined microenvironments in cultural heritage (Liu et al., 2018a; Wang et al., 2025). Therefore, managing humidity is critical not only for prevention, but also for potentially shifting microbial activity toward less harmful states. Finally, preservation strategies, including illumination control and environmental stabilization, have proven effective in mitigating biodeterioration in mural paintings at the Positano Roman Archaeological Museum (Antonelli et al., 2024).

Resistance genes, including multidrug efflux systems and detoxification enzymes, were reported in pigment-producing bacteria from fresco biofilms, where they contributed to the resilience of microbial communities against biocidal treatments (Basile et al., 2025). Interestingly, the genome of *Streptomyces* CH-036 also showed a strong antibiotic resistance profile of genes (Figure 5A). This finding is noteworthy, as certain *Streptomyces* species present in museum environments have been proposed to pose potential risks to the human respiratory system (Borrego et al., 2010). Furthermore, it was pointed out the importance of regular disinfection routines, after several *Bacillus* spp., *Staphylococcus* spp., and *Streptomyces* spp. with potentially pathogenic characteristics were reported in museum collections (Gutarowska et al., 2014; Kraśnicki et al., 2025). It is thus important keep away threats to health from the CHM’s staff and visitors, especially those who are immune-compromised. Protection elements should be used in the manipulation of museum artifacts, and the implementation of disinfection protocols must carefully balance microbial control with material preservation. For instance, ethanol mist has been validated as a biocidal method that significantly reduces microbial load without damaging collagen structures, making it a promising option for delicate heritage materials (Kraśnicki et al., 2025). Also, we encourage the adoption of appropriate measures to avoid new infectious agents coming from the outside of the CHM’s. Proof of the unprotected handling of the book of albumen print is the presence of the commensal species *Staphylococcus epidermis*, related to the human skin microbiome (Villalba et al., 2004). Also, a 99.47% of similarity was found between the genomes of *Oceanobacillus kimchii* CH-031 and *Oceanobacillus kimchii* p3-SID1558 isolated from the human back skin (Table 2).

Some microbes having their optimal growth during the different manufacturing processes of the photo book could have been preserved via spores once the manufacturing conditions changed. Indeed, 7 of the 17 strains from the book identified taxonomically by the VITEK method are producers of endospores (*Bacillus* spp.) or resistance spores (*Streptomyces* spp.). Both endospores and resistance spores are metabolically inactive structures, extremely resistant to adverse environmental conditions, such as high concentrations of salts, absence of nutrients or low levels of humidity. The adaptive advantage conferred on bacteria is to allow them to remain as spores for as long as necessary until the conditions are suitable (Cano and Borucki, 1995; Nicholson et al., 2000; Madigan et al., 2014). Apparently, this could explain the isolation of the thermophilic strain *Caldibacillus thermoamylovorans* CH-041which grows optimally at 50°C, a similar finding previously reported for thermophilic *Geobacillus* spp. in albumen supports (Puškárová et al., 2016). The most likely hypothesis is that the thermophile proliferated during the initial manufacturing (Puškárová et al., 2016). Different methods were used to harden the albumen layers, including steaming and storing the albumen-coated papers inside a warm hayloft for half a year. The temperature of a hayloft can sometimes reach 50°C or more, providing an optimal environment for the growth of CH-041 and other thermophiles (Puškárová et al., 2016). Considering that CH-041 survived for 160 years under environmental conditions far cooler than the ~50°C required for its optimal growth its successful reactivation today suggests a level of genomic stability and preservation over time (Schuenemann et al., 2018; Bonczarowska et al., 2022). A deeper look in to the genomics of CH-041 revealed a scarce amount of genes for antibiotic resistance (Figure 5A), and biodeterioration (Figures 5D,E), making it the most environment friendly strain reported for the CHM.

A particularly intriguing case was observed in Alberdi’s suit, a 19th-century silk and lace garment, where we identified a unique bacterial strain, classified by VITEK as *Actinomyces odontolyticus*. This filamentous bacterium is typically found in human microbiomes, particularly in the oral cavity, and profuselyisolated from dental caries (Batty, 1958). Manifestations of infection by *A. odontolyticus* include thoracic, abdominal, pelvic and central nervous system disease (Razok et al., 2022). Recently, it was proposed as driver of Colorectal Cancer (Breau, 2024). Its presence on the textile suggests potential contamination from human handling or storage conditions, a closed cartoon box that avoids contamination and humidity changes. Notably, the possibility that the original owner of the suit, Juan Bautista Alberdi, introduced this bacterium as a child cannot be ruled out, given the high prevalence of dental infections in toddlers and young children. The genus *Actinomyces* sp. was previously cited in a list of the most common bacteria responsible for biodeterioration of cultural heritage (Grabek-Lejko et al., 2017). A more detailed study of the strain, including genome sequencing should reveal with certainty its taxonomic identity and toxicity, considering the hypothesis that it could have originated from an infected person.

The museum’s wooden furniture is affected by frequent procedures of cleaning and disinfection, as well as repairs and preventive actions against wood decay. Frequently, the wooden material in the museum is sensible to other pests, such as insects. For this reason, it is not expected to these wooden items to suffer biodeterioration driven primarily by bacteria. In spite of this, the isolates from the wooden parts of the chair, table, window, washing trough, and main access door could have cellulolytic or lignocellulolytic capabilities required for the degradation of wood. The most commonly reported bacteria associated with wood-decay environments are genera such as *Clostridium*, and cosmopolitan taxa such as *Bacillus* (Liu et al., 2018b; Pyzik et al., 2021), both isolated from the aforementioned elements (Table 1). A notable high number of genes related to the breakup of cellulose and hemicellulose were found in the genomes of *Microbacterium* sp. CH-007, *Kocuria* sp. CH-021, and *Microbacterium* sp. CH-015 (Figure 5D), which were isolated from the chair wood, the entrance door and the window, respectively, (Table 1 and Figure 3). This could be supporting some kind metabolic activity over the wooden compounds of the furniture. Moreover, the strongest profiles of metal resistances belong to CH-021 and CH-015, and are probably the reflection of the chemical treatments used over the years to preserve the wood from decay. Most of these wood preservatives recruit toxic metals in their formulas, like copper chromate arsenate (CCA) which was widely used in the past but now restricted due its toxicity (Woźniak, 2022). Finally, SEM micrographs of the washing trough showed the presence of filamentous bacteria probably from the genus *Streptomyces* or related (Figure 2D). Previous studies showed that filamentous actinomycetes are associated with wood decay (Antai and Crawford, 1981; Jayasinghe and Parkinson, 2008), while other claim that their presence in wood could prevent worst scenarios of biodeterioration like those caused by fungi (Mortabit et al., 2015; Cai and Kuo, 2022).

One of the strains we isolated from the chair textile was classified as *Bacillus licheniformis*, a species which is in the list of pathogens infecting humans (Bartlett et al., 2022). *B. licheniformis* was reported in several cases of infection, even in a patient with no history of any immune deficiency (Haydushka et al., 2012). Another intriguing species is *Staphylococcus equorum*, isolated from both the textile chair and the washing trough. *S. equorum* is a commensal and obligate aerobe usually found on the skin of various farm animals, such as horses, dairy cattle and goats (Vermeersch et al., 2023; Reydams et al., 2024; Senoussi et al., 2024). This microorganism probably has colonized the seat and meat washing trough during the nineteenth century, when people of the city interacted routinely with farm animals and used horses as their primary vehicle or cattle as meat source.

Regarding the outdoor environment, which includes the main entrance door and the outside wall, the microorganisms found there are related to the street environment and human activity. For example, the species identified by VITEK as *Bacillus pumilus* CH-018, *Clostridium subterminale* CH-019, *Corynebacterium pseudodiphtheriticum* CH-024, *Turicella otitidis* CH-026, and *Micrococcus luteus* CH-020 are either known human pathogens, or related to the human microbiome (Stackebrandt et al., 1995; Bartlett et al., 2022; Zhou et al., 2022; Ahamad et al., 2023). Furthermore, when ANI comparisons are used the genome of the strain CH-019 shares a 99.32% of similarity with *Bhargavaea massiliensis* Marseille-Q1000 isolated from a urine sample in patients with urinary tract infections (Table 2, Section 3.3). Likewise, ANI showed a 98.71% of similarity between the genomes of CH-021 and *Kocuria* sp. CD08_4, an isolate from the duodenal mucosa of a celiac disease patient (Munish et al., 2017). Finally, CH-017 and CH-027 were both classified by Vitek as *B. pumilus* and *B. megaterium* which have been reported as plant pathogens in other work (Bull et al., 2012; Yuan and Gao, 2015).

The genomic profiles of some of the isolates reveal a promising biotechnological potential beyond their role in biodeterioration. Several *Bacillus* and *Pseudomonas* strains identified in this study have previously been proposed as candidates for bioprecipitation of calcium carbonate crystals, a process with practical applications in biorestoration and consolidation of stone and plaster monuments. Notably, *Bacillus megaterium* and *Bacillus simplex*, both detected among the isolates, are among the most effective carbonate bioprecipitators and are used in protective treatments for historical architecture (Paramo-Aguilera, 2012; Pyzik et al., 2021). In addition, members of the Bacillus genus have been suggested for use in biotreatments of textiles and paper artifacts, particularly for their enzymatic capabilities in cleaning and stabilizing cellulosic and proteinaceous materials. Similarly, *Micrococcus* spp. are known for their capacity to produce bioactive molecules and enzymes of interest to the pharmaceutical and bioremediation industries, including antimicrobial compounds and pollutant-degrading enzymes (Díaz-Herráiz, 2015).

Importantly, our genomic analyses revealed that *Streptomyces* sp. CH-036 and *Kocuria* sp. CH-021 harbor genes involved in the synthesis of valuable secondary metabolites and the degradation of xenobiotic compounds (see Figure 5F and section 3.4). These findings position these strains as promising sources of novel biotechnological resources and reinforce the concept of cultural heritage environments as overlooked reservoirs of useful microorganisms. Further exploration of these isolates could yield new applications in conservation, biotechnology, and environmental remediation.

While our investigation prioritized the culturable fraction of the bacterial microbiome, primarily due to its accessibility and relevance for subsequent genomic and phenotypic analyses, it is important to recognize that fungal organisms also play a pivotal role in the biodeterioration of cultural heritage biological origin (Zhgun et al., 2020; Antonelli et al., 2024; Stratigaki et al., 2024; Kraśnicki et al., 2025). Fungi are well-documented agents of degradation, particularly in cellulose- and protein-rich substrates, where their enzymatic capabilities enable persistent and often aggressive colonization. However, characterizing fungal communities typically requires distinct culturing conditions, longer incubation times, and complementary approaches such as ITS-based metabarcoding or cultivation on specific mycological media. Considering these methodological divergences, we prioritized a focused bacterial analysis in this first stage. Future studies could expand the scope by integrating fungal community profiling, thereby offering a more comprehensive understanding of the microbial consortia contributing to heritage deterioration.

## 5 Concluding remarks and prospect

This study provides the first comprehensive microbiological survey of the Casa Histórica de la Independencia Museum, revealing a rich and complex microbial landscape shaped by historical, material, and environmental factors. Through the integration of scanning electron microscopy, microbial isolation, MALDI-TOF MS, and genome sequencing, we uncovered distinct bacteria inhabiting both outdoor and indoor surfaces of cultural heritage significance. This work advances the field of heritage microbiology and supports efforts to safeguard culturally significant objects through a deeper understanding of their microbial ecosystems. Future work should expand to metagenomic and metatranscriptomic approaches, allowing for a deeper exploration of microbial dynamics, functional activity, and long-term succession on heritage substrates.

## Data Availability

The datasets presented in this study can be found in online repositories. The names of the repository/repositories and accession number(s) can be found at: https://www.ncbi.nlm.nih.gov/, bioproject accession number PRJNA1233209.

## Footnotes

^1^ https://sketchfab.com/3d-models/museo-nacional-casa-historica-5b37f88491c0456bbd15fe87f4b8b171

^2^ https://www.novogene.com/amea-en/

^3^ https://doi.org/10.5281/zenodo.15169295

^4^ https://www.genome.jp/kegg/mapper/reconstruct.html
